# Rapid, in-field deployable, avian influenza virus haemagglutinin characterisation tool using MinION technology

**DOI:** 10.1038/s41598-022-16048-y

**Published:** 2022-07-13

**Authors:** Ellen M. de Vries, Noel O. I Cogan, Aneta J. Gubala, Peter T. Mee, Kim J. O’Riley, Brendan C. Rodoni, Stacey E. Lynch

**Affiliations:** 1 Agriculture Victoria, AgriBio, Centre for AgriBioscience, Bundoora, VIC 3083 Australia; 2 School of Applied Systems Biology, La Trobe University, Bundoora, VIC 3083 Australia; 3 Land Division, Defence Science & Technology Group, Fishermans Bend, VIC 3207 Australia

**Keywords:** Influenza virus, Computational biology and bioinformatics, Genomics

## Abstract

Outbreaks of avian influenza virus (AIV) from wild waterfowl into the poultry industry is of upmost significance and is an ongoing and constant threat to the industry. Accurate surveillance of AIV in wild waterfowl is critical in understanding viral diversity in the natural reservoir. Current surveillance methods for AIV involve collection of samples and transportation to a laboratory for molecular diagnostics. Processing of samples using this approach takes more than three days and may limit testing locations to those with practical access to laboratories. In potential outbreak situations, response times are critical, and delays have implications in terms of the spread of the virus that leads to increased economic cost. This study used nanopore sequencing technology for in-field sequencing and subtype characterisation of AIV strains collected from wild bird faeces and poultry. A custom in-field virus screening and sequencing protocol, including a targeted offline bioinformatic pipeline, was developed to accurately subtype AIV. Due to the lack of optimal diagnostic MinION packages for Australian AIV strains the bioinformatic pipeline was specifically targeted to confidently subtype local strains. The method presented eliminates the transportation of samples, dependence on internet access and delivers critical diagnostic information in a timely manner.

## Introduction

The avian influenza virus (AIV) is highly contagious and can cause a globally significant disease of both farmed poultry and wild bird populations with the capacity for human transmission and subsequent pandemic potential. The disease manifests in the respiratory tract of avian species^1^ which, in severe cases can cause widescale death, particularly in poultry farms where birds are in confined spaces, allowing easy transmission of the virus^2^. Single incursions of the disease can therefore spread rapidly within and between poultry farms^3^, resulting in any detections of the virus leading to strict quarantine measures, mass depopulation and decontamination of the area in order to stop the spread^4^. Reports from the World Organisation for Animal Health (WOAH formally OIE) estimated in 2007 that the direct costs of an epornitic would be over 42 billion USD and total indirect costs at 1.5 trillion USD^5^. In more recent years, an agricultural outbreak of AIV in 2014–2015 in the United States of America caused an economic impact of approximately 874 million USD^6^ and more recently, a concurrent outbreak of three AIV strains in Victoria, Australia resulted in the culling of over 400,000 poultry birds in less than six months^7^. These two examples, plus modelling from the OIE, demonstrate the need for proactive detection in farmed poultry and monitoring of virus in wild waterfowl^8,9^.

The disease is caused by infection with an avian derived, influenza virus (species *Influenza A virus,* family *Orthomyxoviridae* genus *Alphainfluenzavirus,*). The AIV genome consists of single-strand negative-sense RNA represented over eight distinct gene segments, contained within an enveloped virus particle^2,10^. The virus is categorised by two glycoproteins involved in virus cell entry and exit, encoded by the haemagglutinin (HA) and neuraminidase (N) gene segments. There are 18 (HA) and 9 (N) different subtypes^2,11,12^. Of the 18 HA subtypes, types H1-16 are present in wild bird populations; the virus’ natural reservoirs, with H17 and H18 exclusively found in bats^11,12^. Disease in farmed poultry and wild bird populations has been reported for many HA subtypes^13^.

The H5 and H7 haemagglutinin subtypes in particular can cause significant disease in poultry^14^ and have been linked to major mortality events in chickens, and other farmed poultry^15,16^. On occasion, zoonotic transmission has also been reported in humans. These two subtypes of AIV cause serious damage in poultry due to the presence of a specific cleavage site in the HA segment^17–20^. In low pathogenicity avian influenza viruses (LPAIV), they contain a monobasic cleavage site. This site is recognised by trypsin-like proteases which are responsible for the cleavage of the HA protein precursor to support virus infectivity and restricting viral infection to the respiratory and intestinal tract^19^, so disease is often mild to moderate in farmed poultry^13^. In high pathogenicity avian influenza viruses (HPAIV), mutations in HA gene segments cause the HA protein precursor to contain a multibasic cleavage site that can be cleaved by ubiquitously expressed furin and other closely related proteases which facilitates systemic spread and entry into multiple cell types, causing higher mortality in poultry.

Within wild bird populations, species within the orders Anseriformes (waterfowl) and Charadriiformes (shorebirds) are considered the main reservoirs^9,14^. Migratory birds within these orders travel through various flyways across the world, with the ability to spread AIV across geographically distinct regions. One example of this being the East Asia Australian Flyway, stretching from far north Russia to Australia^21^. Due to the distance, the birds undertake the journey in stages and therefore have contact with local avian wildlife and poultry, which can lead to transmission of viruses, introducing potential infection to naïve local birds^22^. AIV detections in Australia are generally considered to be genetically distinct from North American and European strains and therefore ongoing monitoring of viruses in local populations provides a baseline for any introductions of international novel influenza strains.

Monitoring of AIVs in wild bird populations is conducted world-wide and is undertaken to understand genetic diversity and ecology of circulating strains, while ensuring diagnostics are fit for purpose to support commercial poultry industries as well as animal, and human health^4^. Protocols for AIV surveillance programs within wild bird populations currently have inherent delays due to sample transport to laboratories and the molecular testing processes used. Currently, pathotype classification (HPAIV and LPAIV) requires in vivo and Sanger genomic sequencing information, which is both slow and expensive. Revised in-field approaches and improved assays would enable faster diagnoses to be made, resulting in better disease containment for the poultry industries^8,14,25^. Whole genome sequencing provides the ability to accurately subtype and identify AIV strains, especially important for H7 and H5 strains where determination of the cleavage site will inform the pathogenicity. Current pathogenicity testing relies on 1st and 2nd generation sequencing (i.e., Sanger and Illumina, respectively), which take a minimum of 24 h to generate the necessary results to deliver the diagnosis; in outbreak settings this sequencing lag time could have compounding effects on the spread of the virus.

The rise of 3rd generation sequencing (i.e., long-read) has enabled fast, on-location, real-time sequencing of whole viral genomes as a viable option for surveillance and outbreak tracing^26–30^. Pioneered by Oxford Nanopore Technologies (ONT), the MinION, a small hand-held portable sequencer, allows remote real-time sequencing of samples out of the laboratory. Direct sequencing eliminates the need to transport samples for testing, which reduces the time to a diagnosis and any potential for sample degradation. Rapid detection and response are of the upmost importance for containing viral outbreaks and supporting control measures, as illustrated in the Zika virus outbreaks in South America^28^. Many studies have progressed the application of molecular screening for viruses outside of standard laboratories in order to utilise the benefits that come with onsite diagnostics^27,28,30–37^. These studies still face the associated challenges encountered with processing samples away from gold-standard laboratory-based facilities. Specifically, extracting nucleic acids and sequencing without cold chain, with minimal power requirements and subsequently undertaking comprehensive bioinformatics, has proved challenging. Although in-field pathogen detection of viruses has been shown to be possible, many of the challenges were not fully overcome and elements of mains connected electrical power or sophisticated cloud bioinformatics were required^31,33,35^. Several studies have also utilised reference mapping-based approach for consensus generation^34,37^, which is not suitable to all viruses, especially segmented viruses with high levels of variation such as AIV.

**Figure 1:**
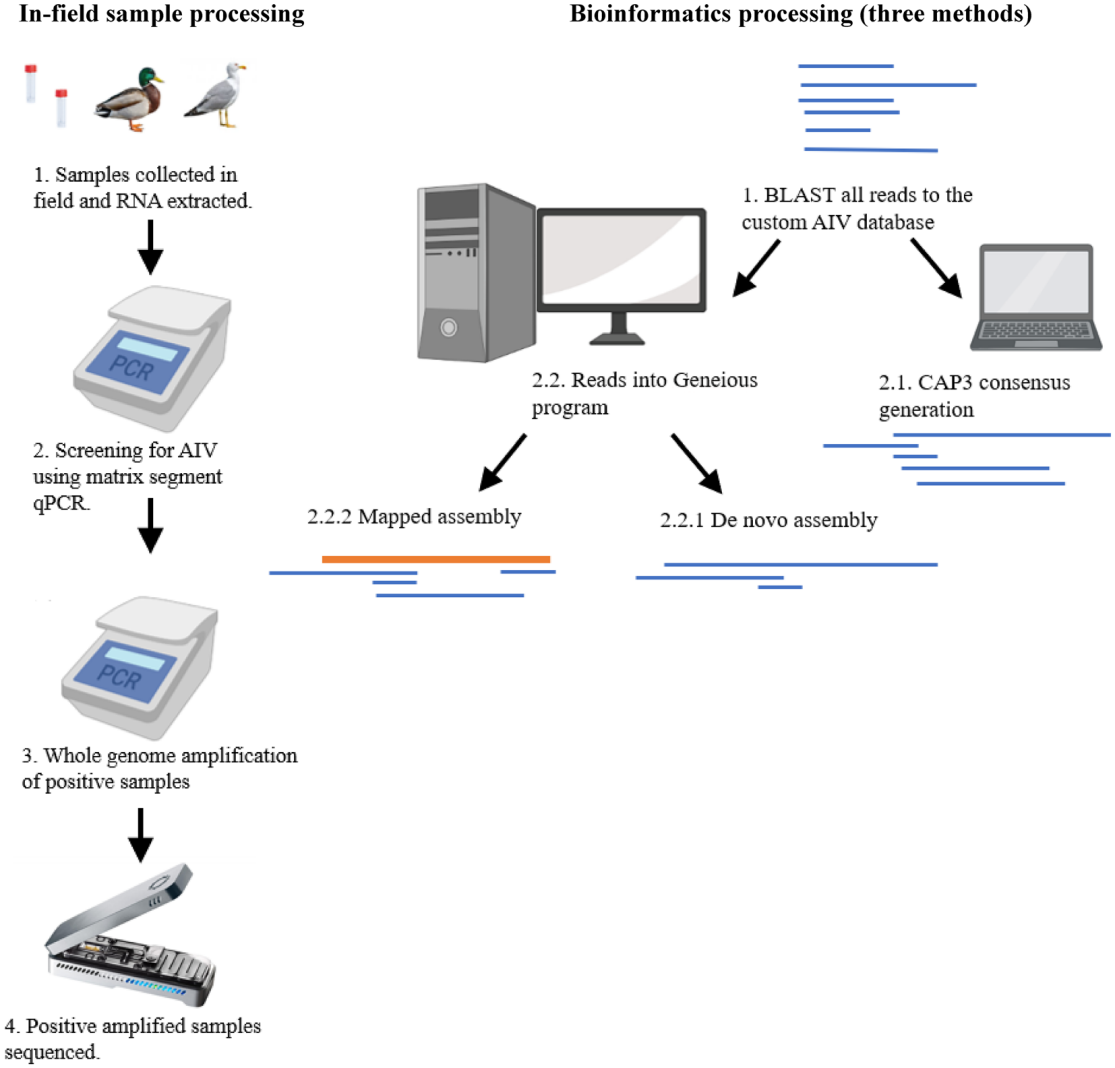
Flow chart of methods used in the study from sample collection to bioinformatic processes.

This study aims to develop an end-to-end in-field protocol and bioinformatic pipeline to enable the diagnostician to go from sample to result (Fig. 1), including nucleic acid extraction, detection, sequencing and bioinformatic analysis of AIV samples collected from Anseriformes and domestic poultry with an emphasis on the classification of the HA segment in minimal time. The specific aim is not to develop a revised perfect diagnostic assay but to develop a HA subtyping surveillance tool that can confidently detect and subtype the HA gene in field. The process was developed to perform RNA extraction and PCR detection of AIV using instrumentation that can be powered from a vehicle, and to complete the sequencing and bioinformatic analysis with no internet access and only a laptop computer and a local preformatted database. Additionally, this study compares MinION derived and Illumina derived sequences to examine inherent error rates and validate and benchmark the accuracy of the newly developed assay against current standards and to assess the use of an influenza MinION positive sequencing control to monitor variation between MinION runs and assess variation in MinION derived sequence reads.

## Materials and methods

### AIV positive and negative samples

Samples used in this study consisted of material collected from wild waterfowl and commercial chicken holdings in Victoria, Australia between 2010 and 2020. Three categories of samples were used in the study: historical AIV positive samples from wild waterfowl (n = 13), commercial poultry (n = 1) and wild bird field samples (n = 36) of unknow AIV status. AIV positive samples consisted of a collection of 13 AIV historical positive samples assembled from stored − 80 °C repositories at Agriculture Victoria and were used in the database. MinION sequencing was performed on a subset (n = 4) of the historical samples and consisted of subtypes H13, H16, H9 and H10. AIV historical samples, subtypes H9 and H10, consisted of cloacal swab samples collected from hunter shot ducks (Lake Bolac, Western District Region, Victoria) from 2019. AIV historical sample, subtypes H13 and H16, were environmental faecal swabs collected from the Werribee Water Treatment Plant (Werribee, Melbourne, Victoria, − 37.98941, 144.62386) in 2019. In addition, a further 36 fresh Anseriformes environmental wild bird faecal samples (of unknown AIV status) were also collected at the Werribee Water Treatment Plant as part of the National Avian Influenza in Wild Bird (NAIWB) surveillance program (wildlife permit 10008927, Department of Environment, Land, Water and Planning, State Government of Victoria) for testing of the in-field deployable laboratory in June 2020. A single commercial chicken sample consisted of pooled faecal material collected from a commercial property during an outbreak of highly pathogenic (H7 subtype) AIV in Victoria in July 2020. All collected swabs were placed in 2 mL of viral transport media (VTM, comprised of brain–heart infusion (BHI) broth containing 2 × 10^6^ U/L penicillin, 0.2 mg/mL streptomycin, 0.5 mg/mL gentamicin and 500 U/ml amphotericin B) transferred chilled at 4 °C to the laboratory, tested within a week of collection and stored at − 80 °C prior to subtype analysis and whole genome analysis.

### Australian AIV HA sequence database

A customised HA gene reference database was developed for the in-field detection and characterisation of AIV in Australia (Table 1). Sequences were obtained from NCBI or GISAID (https://www.gisaid.org/) using the following search criteria: “Influenza database, full length, Australian with the selected HA subtype chosen” (Table 1). The 13 historical AIV samples were also sequenced as part of this study with the sequences deposited in NCBI (OL661617, OL661619, OL661620, OL661621, OL661622, OL661623, OL661624, OL661625, OL675252).

**Table 1:** Viral strains and sequences used in the study divided into custom Australian specific database for HA segment identification and exotic sequences used in the phylogenetic analysis. All viruses are denoted with common nomenclature detailing influenza virus, sample host, location and year.

A: Australian Influenza A screening database		
Subtype	Description	Accession number/Reference
H1	A/duck/Victoria/23/1981(H1N1)	CY077677
	A/Grey_Teal/Victoria/19-1749-184/2019(H1N5)	OL675252
H2	A/mallard/New Zealand/449-81/2004(H2N3)	CY077529
H3	A/wild duck/Victoria/10-03507-020/2010(H3N8)	Wille et al.^66^
	A/Pink-eared Duck/Victoria/PD001/2017(H3N1)	MK213331
H4	A/Chestnut_Teal/Victoria/19-1749-226/2019(H4N6)	OL661622
	A/Chestnut_Teal/Victoria/19-1749-32/2019(H4N6)	OL661623
H5	A/duck/Victoria/0305-2/2012(H5N3)	CY111533
	A/duck/Victoria/26/1981(H5N2)	CY077685
	A/shearwater/Australia/751/1975(H5N3)	CY045255
H6	Pacific black duck/Western Australia/1980(H6N8)	CY077660
	A/Eurasian coot/Western Australia/2727/1979(H6N2)	CY028243
H7	A/starling/Victoria/1/1985(H7N7)	M17736
	A/chicken/NSW/1/1997(H7N4)	AY943924
	A/chicken/Victoria/1/1992(H7N3)	AF202227
	A/chicken/Queensland/667/1995(H7N3)	AF202231
	A/duck/Victoria/512/2007(H7N6)	CY061610
	A/duck/Tasmania/277/2007(H7N2)	CY033161
	A/chicken/Victoria/20-02865-0008/2020(H7N7)	OL661625
	A/Grey Teal/Victoria/19-01749-185/2019(H7N8)	Wille et al.^66^
H8	A/duck/Bangladesh/37525/2019(H8N4)	MT090367
	A/duck/Hokkaido/X9/2016(H8N4)	MK978904
H9	A/Grey Teal/Victoria/GT001/2017(H9N1)	MK213322
	A/Grey_Teal/Victoria/19-1749-185/2019(H9N4)	OL661624
H10	A/Grey_Teal/Victoria/19-01749-193/2019(H10N8)	Wille et al.^66^
	A/Chestnut_Teal/Victoria/18-01589-268/2018(H10N7)	OL661617
H11	A/wild_waterbird/Victoria/19-0581-09/2019(H11N9)	Wille et al.^66^
	A/sharp-tailed sandpiper/Australia/6/2004(H11N9)	DQ327835
H12	A/Influenza A Virus/Australia(H12N5)	MH453831
	A/red-necked stint/Western Australia/5745/1982(H12N9)	CY094879
H13	A/wild_waterbird/Victoria/19-4759-4/2019(H13N8)	OL661619
	A/wild_waterbird/Victoria/19-4759-3/2019(H13N8)	OL661620
H14	A/Blue-winged_Teal/Ohio/18OS1695/2018(H14N5)	MN431050
	A/goose/Karachi/NARC-13N-969/2014(H14N3)	KX602668
H15	A/sooty tern/Western Australia/2190/1983(H15N9)	CY006033
	A/Australian shelduck/Western Australia/1756/1983(15N2)	CY006032
H16	A/wild waterbird/Victoria/19-04759-011/2019(H16N3)	Wille et al.^66^
	A/wild_waterbird/Victoria/19-4759-7/2019(H16N3)	OL661621
H17	A/little yellow shouldered bat/Guatemala/060/2010(H17N10)	CY103892

B: Exotic sequences used in phylogenetic analysis		
Subtype	Description	Accession number
H1	A/duck/Yuhuan/YH45/2016(H1N2)	KY415627
H2	A/Pigeon/Longquan/LQ67/2016(H2N8)	KY415628
H3	A/duck/Fujian/SD063/2017(H3N3)	MG204059
H4	A/duck/Ganzhou/GZ5/2015(H4N6)	KY415636
H5	A/duck/Hubei/SZY250/2016(H5N1)	KX523694
	A/goose/Guangdong/1/1996(H5N2)	AF144305
H6	A/duck/Fujian/SD099/2017(H6N6)	MG198927
H7	A/duck/Guangdong/1/1996(H7N3)	JQ988864
	A/chicken/China/AS1/2019(H7N9)	MN700033
H8	A/duck/Yangzhou/02/2005(H8N4)	EF061122
H9	A/chicken/China/1104/2019(H9N2)	MN918143
H10	A/chicken/Zhejiang/516,100/2017(H10N3)	MG366507
H11	A/duck/Fujian/SD061/2017(H11N3)	MG214160
H12	A/Mareca_falcata/China/D29/2015(H12N2)	MK301259
H13	A/Duck/China/Weihai/2017(H13N8)	MH894208
H15	A/duck/Bangladesh/24,697/2015(H15N9)	KY635719
H16	A/Herring Gull/Delaware/597/2019(H16N3)	MN911206

Where no Australian AIV HA subtype sequences could be found using the above database text searches and were absent from the Agriculture Victoria historical collections, southern hemisphere full length sequences from countries of the East Asia Australia Flyway^14^, or neighbouring countries were used. HA segment sequences for the H8 and H14 subtypes were identified as absent and two sequences for each subtype were included in the database (Table 1).

### Illumina based genomic sequencing

Whole genome Illumina based sequencing was performed on the 13 historical swab samples to enhance the AIV Australian database and for experimental MinION comparison (n = 4). RNA was extracted using QIAamp Viral RNA Mini Kit (Qiagen, Hilden, Germany), whole genome amplified^38,39^ and Illumina sequencing libraries prepared using PerkinElmer NEXTFLEX Rapid Directional RNA-Seq Kit 2.0 (Perkin Elmer, Waltham, MA, USA). The finished libraries were quantified using a dsDNA HS assay on the Qubit (V3) (ThermoFisher Scientific, Waltham, MA, USA) and an HSD1000 assay on the Tapestation 2200 (Agilent, Santa Clara, CA, USA), pooled in equimolar concentrations and diluted to 7 nM. The libraries were sequenced using an S4 NovaSeq flow cell or a MiSeq 600 cycle V3 kit. The sequences were assembled through IRMA^40^, an iterative assembler built for influenza construction, using the default FLU parameters.

### Synthetic AIV positive control for RT-PCR and MinION sequencing

A positive control was included to monitor the inter-assay performance of AIV whole genome amplification, the MinION DNA library preparation and subsequent sequencing. The positive control consisted of a synthetic double stranded DNA (dsDNA) fragment (Gene block, Integrated DNA Technologies, Iowa, USA) encoding the H17 HA gene segment (Genbank accession number CY103892.1 bp 1–1784), flanked at either end by the influenza universal whole genome amplification primer sequences (MB-Tuni12 and 13 primers)^41^. This enabled amplification of the synthetic positive control along with the AIV samples. A mass of 40 pg of the dsDNA gene fragment was amplified with 45 µL Platinum PCR High Fidelity SuperMix (1U enzyme) (ThermoFisher, MA, USA), and 1 µL of 10 µM solution of each MB-Tuni12 and MB-Tuni13^41^ primers in a total volume of 50 µL. The mix was amplified with the following conditions: 94 °C for 30 s then 30 cycles of 95 °C for 30 s, 55 °C for 30 s and 68 °C for 2 min. The amplified product was purified using an Isolate II PCR and Gel Kit (Bioline, London, UK), diluted to 1/1000 and quantified using the Qubit (V3) HS dsDNA kit (Thermo Fisher Scientific, MA, USA). The cleaned, amplified dsDNA positive control was diluted to form three solutions of varying copy number: (1000, 10,000 and 100,000) based on calculations using the molecular weight and Avogadro’s number^42^. The 1000 copy number solution was then used as a positive control in all batches of samples to monitor reproducibility of the PCR and MinION sequencing procedures.

### In-field RNA extraction method and laboratory comparison

RNA from all samples in this study were extracted using the in-field Biomeme M1 Bulk Sample Prep Kit (Biomeme Inc., Philadelphia, USA). Optimisation for AIV detection required major modifications to the standard protocol. Briefly, 140 µL of the samples in VTM was mixed with 280 µL of Biomeme lysis buffer by carefully drawing the liquid mixture up and down approximately 30 times in the provided syringe with a silica membrane. The liquid was incubated in the syringe for two minutes and expelled (removing all liquid). The syringe was washed with one pump of 500 µL of the Biomeme Protein Wash followed by 750 µL of either the Biomeme Wash Buffer, or the Invitrogen PureLink Viral RNA/DNA wash buffer II (Thermo Fisher Scientific, MA, USA), which was found to be equivalent (data not shown). A 1000 µL aliquot of the Biomeme Drying Wash solution was drawn up and expelled and the syringe air dried by repeated drawing up and expulsion of air for approximately 30 pumps until no droplets were expelled. The syringe was left to air dry for one minute before 150 µL of the Biomeme Elution Buffer was drawn up into the syringe and left to incubate for two minutes at ambient temperature. The liquid was expelled and drawn up for seven pumps before final expulsion of the eluate into a clean 1.5 mL tube. The resulting 150 µL nucleic acid extract was used for testing and sequencing.

RNA was also extracted using the Invitrogen PureLink Viral RNA/DNA (Thermo Fisher Scientific, MA, USA) following the manufacturer’s instructions. Comparative analysis between the Biomeme M1 and Invitrogen PureLink Viral RNA/DNA spin extraction kit used serial dilutions of AIV-infected cell lines which were spiked into negative field collected, environmental faecal material and the relative viral load was determined by RT-qPCR.

### In-field AIV screening of nucleic acid extracts using RT-qPCR

Nucleic acid extracts were tested for AIV by RT-qPCR which amplifies the matrix segment of the virus. Briefly, samples were processed following the AIV Type A generic assay^38^ with 1 µL of the primer/probe mix (containing 0.9 µM of both forward and reverse primers and 0.25 µM of probe from Heine et al.^38^), 5 µL of extracted RNA, 12.5 µL of X2 AgPath-ID One Step PCR RT buffer, 1 µL of X25 AgPath-ID RT PCR enzyme (Thermo Fisher Scientific, MA, USA) and molecular grade water to make up a 25 µL volume. The samples were then run with the following conditions: 45 °C for 60 min; 95 °C for 10 min; and 45 cycles of 95 °C for 15 s, 60 °C for 45 s. For the in-field deployable laboratory, the RT-qPCR assay was conducted on a Mic PCR machine (BioMolecular Systems, New South Wales, Australia) supported by a 12-V battery (Itech 120AH LifePo4) linked to an Itech 2000-W Sinewave inverter and powered by a 100 series 4 × 4 Toyota Landcruiser. AIV positive samples were taken forward for sequencing.

### In-field protocol for whole genome amplification of AIV

Whole genome amplification of the AIV positive samples was performed as described^38,39^ with only 20 cycles of routine amplification. Briefly, samples were mixed with 12.5 µL Superscript III One-Step PCR reaction buffer, 0.5 µL of SuperScript III RT/Platinum Taq Mix (Thermo Fisher Scientific, MA, USA), 0.5 µL of each individual primer (MB-Tuni12 and 13^41^), 2.5 µL of RNA template and water to make up 25 µL volume. The samples were then run with the following conditions: 42 °C for 60 min, 94 °C for 2 min, 5 cycles of 94 °C for 30 s, 45 °C for 45 s, 68 °C for 3 min, and 20 cycles of 94 °C for 30 s, 57 °C for 45 s, 68 °C for 3 min and a final extension of 68 °C for 5 min.

### In-field protocol for library preparation

Libraries for sequencing were prepared from the whole genome amplified positive samples (7.5 µL per sample) using the ONT Rapid Barcoding Kit (SQK-RBK004, protocol version RBK_9054_v2_revN_14Aug2019) with modifications. No magnetic bead clean-up was used after barcode pooling and 1.3 µL of RAP was used in the 13 µL library. 1.2 µL of nuclease free water was added in the final library preparation step instead of the recommended 4.5 µL as the pooled barcoded samples was 14.3µL Approximately 500 copies in 5.5 µl of the H17 amplified gene segment made up the dsDNA positive control. The negative control was a sample from a previous surveillance trip that tested negative that was carried through the same process as the positive samples. Each sample had unique DNA barcodes ligated and were then pooled to form a single sequencing library. The volume of each sample pooled for library preparation was adjusted according to the Ct detected using the AIV RT- qPCR, with post-amplification values ranging from 6.5 to 16.6. Nucleic acid extracts with a Ct of 20 and above had 2.5 µL of the whole genome amplified material added to the final pooled library. A sample with a Ct value in the range of 10–15 had 1.5 µL added to the library and a sample with a Ct value of less than 10 had 1.0 µL added. Bead cleaning was not performed and the ratio of rapid sequencing adaptors (RAP) to pooled barcoded DNA was maintained (1.3 µL for 13 µL of pooled DNA). The final pooled DNA library was diluted to a total volume of 15.5 µL.

### Development of a deployable, in-field avian influenza virus detection and characterisation system

**Figure 2:**
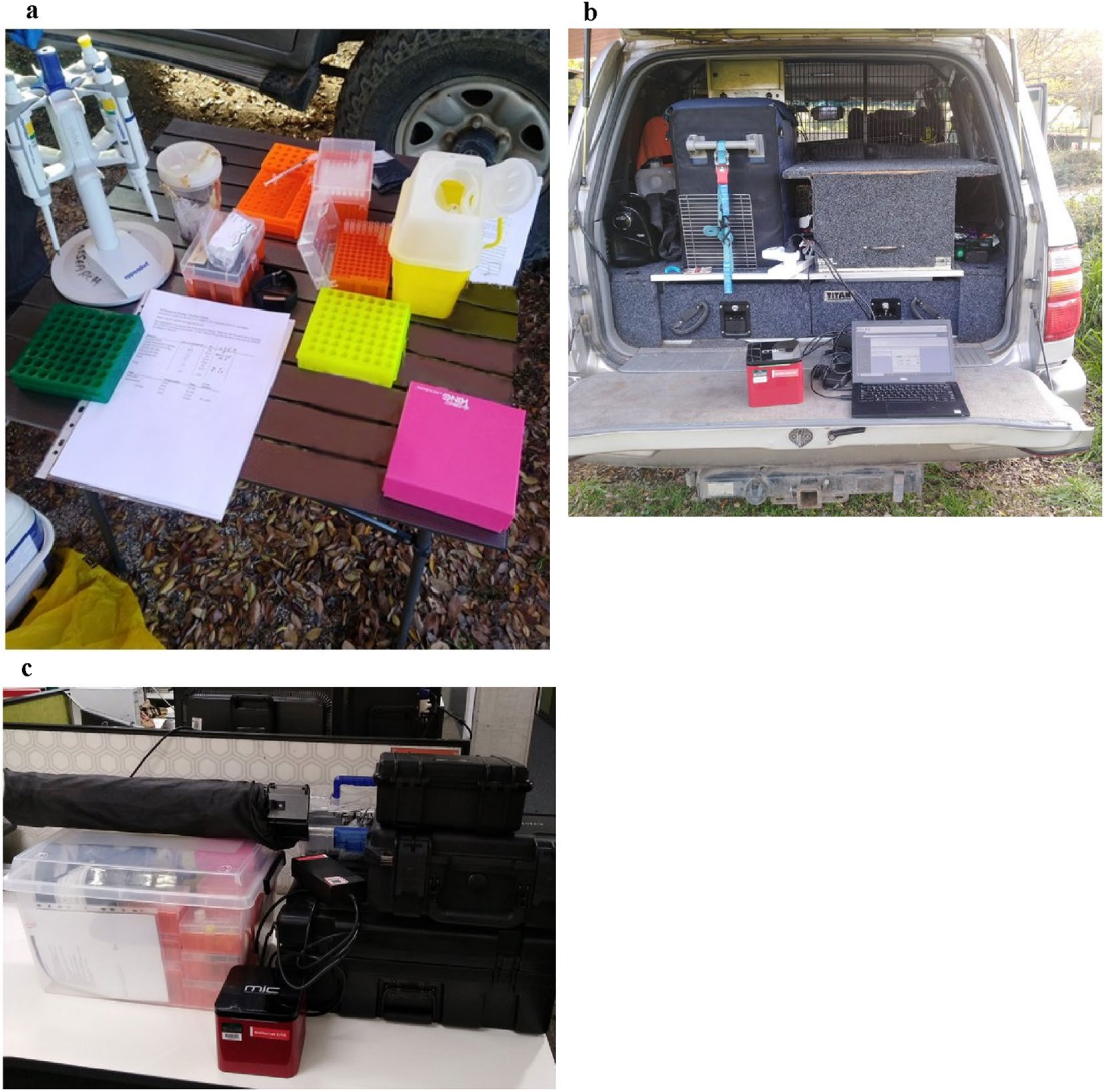
Deployed and functional portable laboratory set up. (**a**) Mid-RNA extraction in-field using the Biomeme M1 RNA extraction kit. (**b**) The MIC PCR machine performing that TaqMan assay out the back of the Landcruiser. (**c**) The entire kit packed up (all equiptment listed in Supplamentary data 1).

The portable system consisted of three main components- (1) wet lab reagents and consumables (Supplementary data 1), (2) the Mic PCR machine for AIV screening of samples and whole genome amplification of positive samples, (3) and the portable sequencer (MinION Mk1B device) and associated high-powered laptop. A minimal number of boxes and equipment were used to transport equipment needed to set up a small diagnostic-like laboratory. Everything required for the analysis in-field was packed up and transported in a single box (Fig. 2a–c). The Dell Precision laptop was transported in a pelican case which had specific foam indents for the MinION Mk1b to be stored in. A small camping table was also bought alongside.

### MinION sequencing and real-time, offline basecalling

The pooled barcoded MinION library was loaded onto the MinION Mk1b (Oxford Nanopore Technologies, Oxford, United Kingdom) sequencer with an R9.4 flow cell. The sequencer was powered by a custom Dell Precision 7740 laptop (64 GB SD RAM, 4 TB SSD, Intel® Xeon 8 Core Processor, Ubuntu 18.04, NVIDIA Quadro RTX 5000 GPU). The library was basecalled in real-time using MinKNOW version 3.0.4 with offline basecalling (Guppy version 3.2.10) enabled so to be independent of any internet connection requirements (provided by Oxford Nanopore upon request). Basecalled reads were converted from fastq to fasta file format using seqtk (version 1.3) (https://github.com/lh3/seqtk) for subsequent analysis.

### AIV HA subtype characterisation

Fasta files from the test samples were BLASTn analysed (version 2.6.0) with the following parameters specified; outfmt 6 -max_target_seqs 1 -max_hsps 1 -evalue 0.00001, against the customised Australian AIV database as part of this study (Table 1) to identify HA subtype which in turn was used for consensus sequence generation.

Three distinct bioinformatic workflows were developed and evaluated to determine the best in-field bioinformatic process for AIV HA subtype characterisation. The following parameters were selected and measured as bioinformatic performance metrics: time and computational power required to complete the analysis; nucleotide similarity (when compared to Illumina generated genomic sequences of the same sample); and HA gene segment coverage. BLASTn 2 Sequences (version 2.6.0) program was used to compare the consensus sequence output for all three methods. Reads that mapped to the Australian AIV database (Table 1) are referred to as the mapping reads and were then processed through the following methods.

#### Method 1- CAP3 de novo assembly

The CAP3 tool for assembly and contig construction was evaluated, which uses nucleotide overlaps in reads to construct contigs^43^. Generated contigs were then assembled into an HA segment consensus sequence. Subsequently, the top two contigs, or the singular assembled consensus sequence (whichever was longer), were analysed using BLASTn. A BLASTn 2 sequence analysis was manually run locally with the generated consensus sequence and sequence which it mapped to from the database. The identity and coverage of the contigs were recorded. The CAP3 de novo assembly pipeline did not interfere with the ongoing sequencing and could be initiated at any time but would only uses the data generated up to that point in time.

#### Method 2- Geneious de novo assembly

MinION reads mapping to the Australian avian influenza HA sequence database were imported into Geneious (Version 2020.1.1) and processed using the de novo assembler with high sensitivity/medium selected as an option. “Trim sequences” and “save contigs” (max 1000) were both selected. The contig with the most mapped reads was used to generate the consensus sequence by selecting the “generate consensus” option with a 25% strictness setting.

#### Method 3- Geneious reference mapping

A second method using Geneious (Version 2020.1.1) was used to produce a HA consensus. MinION reads mapping to the Australian avian influenza HA sequence database were imported into Geneious. The viral Influenza Database subtype with the most corresponding hits was used as the reference and the MinION reads were mapped to the representative subtype sequence. The resulting mapped consensus was used in subsequent analysis.

### Phylogenetic analysis

The custom-made database and method outputs from this study were made into a phylogenetic tree which included avian associated HA subtypes (H1-H16) the existing Australian database, which was enhanced with exotic strains, resulting in 55 sequences in total (Table 1A and B). The sequence data was processed using MEGA11, Maximum Likelihood tree builder, 1000 bootstrapping, nucleotide substitution, Jukes-Cantor Model with partial deletion and the average site cut-off at 51%.

## Results

### Generation of custom database for AIV diagnostics

A collection of 13 historical AIV positive swab samples were identified, whole genome sequenced on an Illumina platform and assembled using IRMA to act as gold standard references. All assembled viral whole genome sequences were HA segment confirmed through online BLAST analysis. These sequences along with other NCBI or GISAID sequences formed the Australian AIV database (Table 1A), resulting in a total of 40 sequences.

### In-field RNA extraction method for AIV detection and sequencing

**Figure 3:**
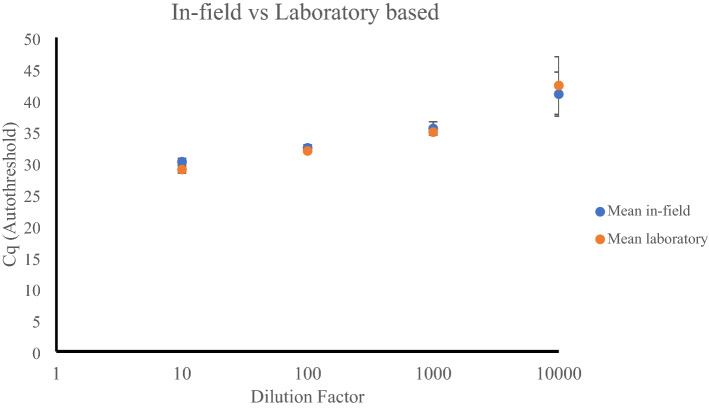
A comparison of in-field Biomeme M1 Bulk RNA Kit (blue) and laboratory based nucleic acid extraction kit (Invitrogen PureLink RNA/DNA extraction kit) (orange) for the recovery of AIV RNA in environmental faecal samples. Mean Cq values from three replicates tested in the AIV RT-PCR assay is shown (total number of samples = 30, three samples per dilution per kit). Error bars represent the standard deviation of experimentation run in triplicate.

An optimised in-field RNA extraction protocol was developed to support the detection and sequencing of AIV from avian cloacal swabs and environmental faecal samples. To assess the recovery of nucleic acid the in-field RNA extraction method (Biomeme M1 Bulk RNA) was compared with the laboratory based nucleic extraction kit (Invitrogen PureLink RNA/DNA extraction kit). The optimised in-field syringe-based extraction method yielded viral RNA levels comparable to the laboratory based nucleic acid extraction kit as determined by AIV specific qRT-PCR (Fig. 3). For AIV dilutions of 1/10 to 1/10 000 the in-field extraction method obtained Ct values of 30.21 (se 0.55) to 40.97 (se 3.51) respectively, compared to 28.97(se 0.57) to 42.35 (se 4.6) for the laboratory based nucleic extraction kit respectively (Fig. 3).

### HA subtype classification

The positive synthetic H17 control included in all batches processed, gave a consistent indication of the quality of MinION runs and therefore whether the subsequent sequence data from field samples could be used. The cut off for a successful run was deemed to be > 100 mapping reads from the synthetic control to the database (data not shown).

A subset of four (H9, H10, H13, H16) samples from the historical AIV positives repository, a commercial poultry sample (H7) as well as the ds DNA H17 positive and the negative control were processed through the in-field MinION HA characterisation tool (Table 2). Generated reads were characterised using a BLASTn comparison to the HA Australian Database generated in this study (Table 1). The majority of samples generated database hits that were identified as a singular predominant HA subtype (H9 = 5202 (99.8%), H10 = 9787 (99.9%), H16 = 227 (86.3%), H17 = 249 (90.5%), H13 = 1533 (98.9%), H7 = 22,503 (100%)). The AIV sample (H16 subtype) that was detected and sequenced had the lowest number of correctly attributed reads with the majority of the reads (34) that were misattributed were designated as H13 (21, (7.9%)). The negative control generated 535,589 reads, with a single read mapping to the AIV Australian database (H9).

**Table 2:** MinION generated reads mapping to the Australia AIV HA database** to enable real-time subtype level classification of reads.

	Total reads generated	Total hits to the HA database**	H1	H2	H3	H4	H5	H6	H7	H8	H9	H10	H11	H12	H13	H14	H15	H16	H17
**H type sequenced**																			
H7*	10 000	22 503	0	0	0	0	0	0	22,503	0	0	0	0	0	0	0	0	0	0
H9	3 391	5210	0	0	0	0	0	0	0	0	5202	2	0	5	0	0	0	1	0
H10	7 491	9793	0	0	0	0	0	0	0	0	0	9787	0	0	0	0	1	0	0
H13	6 067	1549	0	0	0	0	0	0	0	0	0	14	0	0	1533	0	0	2	0
H16	4 001	263	0	0	0	0	0	0	0	0	3	10	0	0	21	0	0	227	0
H17	12 385	275	0	0	0	0	0	0	0	0	3	20	0	0	1	0	0	0	249
Negative	535 589	1	0	0	0	0	0	0	0	0	1	0	0	0	0	0	0	0	0

*H7 was subsampled to 10 000 reads from the total generated reads.

**Refer to Table 1 for sequences.

### HA subtype genomic characterisation

**Figure 4:**
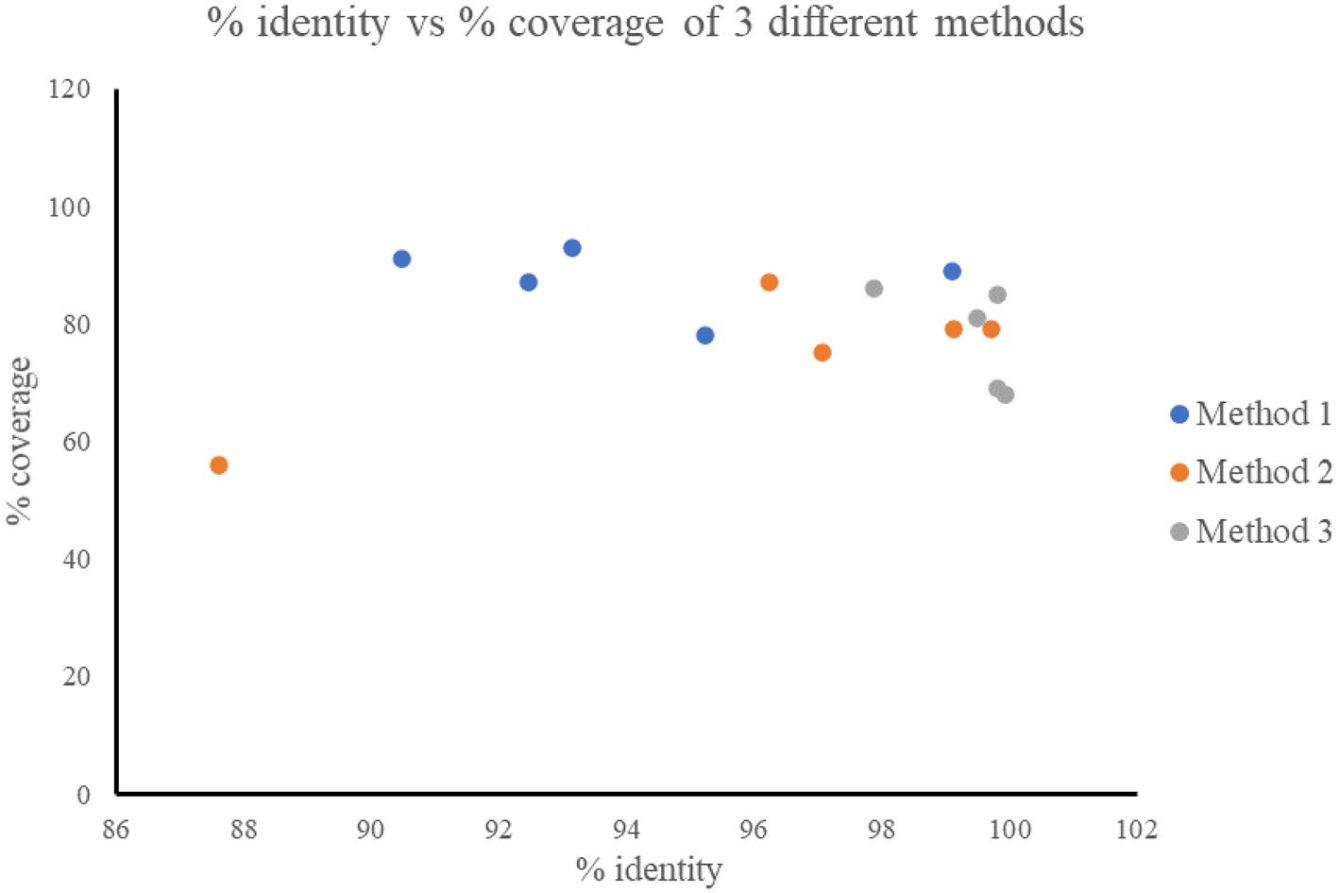
Consensus sequence comparison of three different analyses methods (Method 1- CAP3 de novo assembly. Method 2- Geneious de novo assembly, Method 3- Geneious reference mapping) against Illumina derived reference sequence, measured using BLASTn 2 Sequences program. The Illumina generated IRMA assembled consensus was compared to each of the MinION based consensuses to assess on accuracy.

Following the HA sequence identification, the filtered reads were sub-selected into a new fasta file and assembled through the three methods presented. The pipelines differed in both the computational requirements and time to results (Table 3). A comparison of the percentage sequence identity and percentage coverage of the HA segment of six selected AIV isolates generated by the in-field MinION sequencing method was made with the corresponding Illumina sequences (Fig. 4; Table 3). All of the samples generated consensus sequences that on average had over 75% coverage for the HA gene and were > 90% identical to their relevant Illumina reference sequence (Table 3).

**Table 3:** Comparison of the three different MinION assembly methods (Method 1 CAP3 de novo assembly, Method 2 Genious de novo method and Method 3 Geneious reference mapping) used to assemble MinION generated, BLASTn mapped reads of the AIV HA segment.

Method	Assembly	Output	Time for one barcode	Time investment for 8 parallel barcodes	Mean of % identity	St dev % identity	Mean of % coverage	St dev of % coverage
Method 1 (CAP3)	Overlapping fragments	Consensus from multiple contigs	~ 10 min	~ 30 min	94.09 (90.48–99.11)	2.9	87.6 (78–93)	4.7
Method 2 (Geneious)	De novo assembler	Consensus from contig with most reads	~ 30 min	~ 6 h	95.95 (87.61–99.13)	4.4	75.2 (56–87)	9.5
Method 3 (Geneious)	Reference mapping	Singular consensus	~ 20 min	~ 4 h	99.39 (97.87–99.82)	0.77	77.8 (69–86)	7.1

All reads were parsed through the Australian HA curated database and analysed in comparision with the Illumina reference using BLASTn 2 Sequences.

Across the different subtypes, CAP3 de novo assembly Method 1 generated results within 10 min for a single barcode (single sample), could run multiple barcodes in parallel and showed the highest coverage (mean 87.6%; range 78–93%) across the HA segment, with a high percentage identity (mean 94.09%; range 90.48–99.11%). Geneious de novo assembly Method 2 and reference mapping Method 3 required a longer processing time (up to 4 h), and as neither method could process multiple barcodes at the same time, would take an estimated 4–6 h to process eight barcodes. Method 2 had the lowest coverage (mean 75.2%; range 56–87%) with a percentage identity comparable to Method 1 (mean 95.95%; range 87.61–99.13%), but also had the largest variance of both mean percentage identity and percentage coverage. Method 3 generated the highest percentage identity (mean 99.39%; range 97.87–99.82%), which was consistent across the subtypes as demonstrated by the low standard deviation (0.77) across more than two thirds of the HA segment (mean 77.8%; range 69–86%) (Table 3).

### Phylogenetic analysis of in-field MinION derived consensus sequence

**Figure 5:**
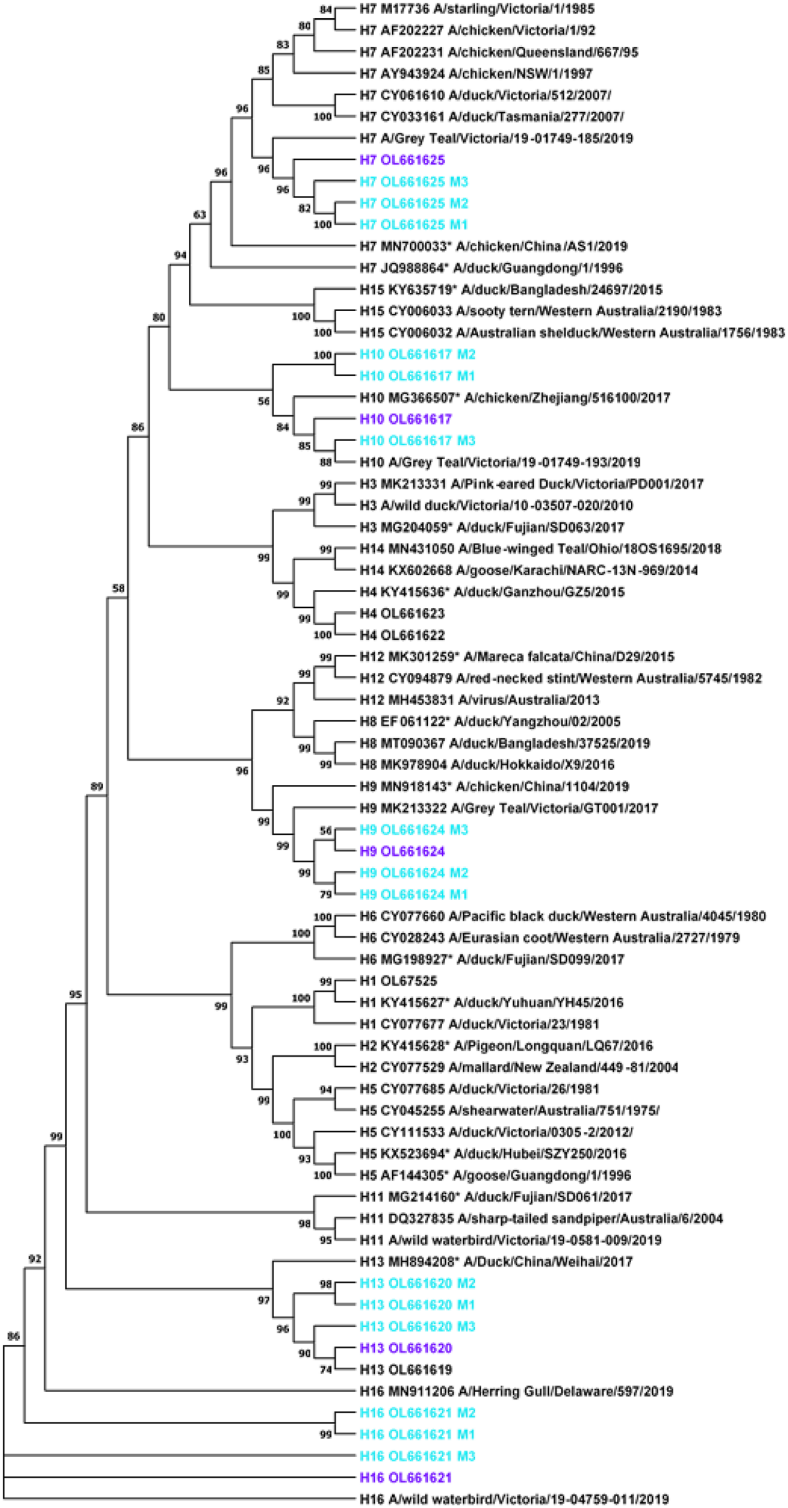
An unrooted maximum likelihood (ML) of 55 avian influenza A virus haemagglutinin sequences (Table1A and B, excluding H17) worldwide obtained from MEGA11. The ML tree shows all 16 influenza subtypes as their own unique clades. Bootstrap values below 50% are not shown. All consensus HA sequences generated in this study are indicated with a light teal colour and the following nomenclature for the different pipeline methods—M1- CAP3, M2- Geneious de novo, M3- Geneious reference mapping. Corresponding Illumina sequences are indicated with purple. Exotic strains are denoted with *. All other strains are Australian unless otherwise stated in the study. Pairwise patristic distance of sequences can be found in Supplementary Data (2).

A comparison of the consensus sequences generated using the three bioinformatic methods was undertaken through phylogenetic analysis that included the sequences from the Australian avian influenza sequence database, in addition to exotic genomic sequences (Fig. 5). The HA subtypes created their own clades with some subtypes creating closer genetic relationships (H13/H16, H15/H7 and H14/H4), suggesting higher degrees of evolutionary relatedness than with other subtypes. The consensus sequences generated using the three bioinformatic methods described in this study, placed the derived genomic sequences within their respective HA subtype branches. Phylogenetic analysis identified the consensus sequences derived using Method 3, most accurately matched the related Illumina derived sequences, (Fig. 5).

### In-field protocol deployment

The in-field deployable sequencing capability (Fig. 2a–c) was successfully tested in Werribee, Victoria. RNA was extracted from 36 field samples and screened for the presence of AIV using whole genome amplification^38,39^. No positive samples were identified from the 36 samples screened as confirmed by the laboratory-based AIV qRT- PCR (data not shown).

## Discussion

Early detection of viruses is critical for managing potentially detrimental impacts of a disease outbreak, as a fast and effective response increases the chances of pathogen containment and eradication^44^. In this study, we describe a field deployable laboratory that can, through a series of functional wet lab and bioinformatic processes, detect AIV in field collected samples as well as characterise and phylogenetically analyse the AIV HA gene sequence, all within hours of sample collection. Although the proposed methods presented here are most likely not at the sensitivity level of standard matrix gene qPCR assays, it is a fast, confirmatory test which allows faster and more specific actions if required.

The rapid development of genomic technologies in the past decade has enabled the possibility of novel, point-of-care genomic diagnostics to become a reality^45^. The in-field sequencing capability described in this paper has been achieved using a range of recently available technologies and reagents including syringe-based RNA extractions, a rapid library preparation kit for in-field ONT sequencing and bioinformatic analysis of datasets. Use of syringe-based extractions has been effective in previous viral studies^46^ and was effective in this study for the detection and amplification of AIV RNA in a strictly in-field setting with extraction results comparable to those generated by laboratory-based RNA extraction protocols (Fig. 3).

Although AIV has been sequenced using the MinION^34,37,47–49^, a field-deployable sequencing method for HA subtype classification, has not been reported. The ONT sequencing protocols using the Rapid Barcoding Kit (SQK-RBK004) have been successful but required lab-based spin column extraction methods, quantification of nucleic acid concentrations with Qubit and magnetic bead clean-up of the pooled sequence libraries that requires extra utilities which increases the equipment footprint of the methods. The protocol for pooling Rapid Kit barcodes recommends that the clean-up of libraries using magnetic beads is required to remove excess adapters and nucleic acids. However, magnetic beads require extra equipment and a sterile environment, which are both impractical for field work and pooling barcodes runs the risk of overloading the MinION flowcell with excess nucleic acid. In order to circumvent these requirements, the Rapid protocol was modified to vary the volume of sample added depending on the Ct value of the AIV samples derived from the qRT-PCR^38,39^ (and therefore the concentration of nucleic acid). This method removes the need for quantification on instruments (i.e., Qubit) but also allows the user to have control over the amount of nucleic acid used.

The ONT Rapid Kit uses transposon-based fragmentation of DNA resulting in short and variable read lengths of moderate quality as the output. These inherent differences mean that the ONT reads generated from the ONT Rapid Kit are not suitable for short read specific de novo assembly programs that are designed specifically for pair end reads generated from Illumina sequencers. Reads generated in this study (ranging from between 160 and 1800 bp) were also too short for conventional MinION de novo assemblers such as wtdbg2^50^, Canu^51^ and Flye^52^. Although the reads were of similar sizes to second-generation high-throughput sequence data, MinION reads do not contain Phred scores and are not paired reads, two points of data which are used in the analysis of second-generation high-throughput sequence data. Consequently, reference-based approaches have been typically used to generate viral consensus sequences from the Rapid Kit^27–30,34,37,53^. More sophisticated methods of HA subtype classification have been developed, for example IRMA, an iteratively trained assembler developed by the Centre for Disease Control (USA). IRMA is a well-accepted and efficient method of genomic characterisation of virus genomes from short read and high quality ONT data that has been widely used^37,48^. However, the AIV database developed for IRMA is built and trained on sequence data of American viruses and strains and given the high variability between HA subtypes and clades this is not currently an appropriate approach for ONT Rapid Kit derived sequence data in Australia.

To overcome these issues and to classify HA subtypes from MinION sequencing reads, a “BLASTn-to-database method” was used in this study. Although similar approaches have been used before^34,47^ the bioinformatic approaches did not have the initial BLAST based analysis step and employed the Method 3 (Geneious) approach tested here, which is impractical for in field deployment given the computational and time (~ 4 h) investment. Reference mapping approaches also require prior knowledge of the samples, an issue when using unknown field samples. McCuen et al.^49^ utilised a BLAST step to identify unknown field samples before lab-based processing using the Influenza Research Database curated by the U.S National Institute of Allergy and Infectious Diseases containing over 10,000 sequences, mostly originating from the Northern Hemisphere. The Influenza Research Database contained only 92 Australian sequences, covering just 11 HA subtypes (missing H16, H13, H14, H8, H2, H17) and only 18 of these sequences were generated post-2000 (19.5% of sequences). The data set assembled and used in this study (Table 1) contains 26 unique sequences post-2000 (69% of sequences in the database, the H5 is identical in both databases) that covered 13 subtypes as well as four subtypes that haven’t been detected in Australia (H2, H8, H14, H17).

The BLASTn-to-database screening method used to identify AIV sequence reads and assign putative HA subtype is effective as there is significant sequence divergence between subtypes of AIV (Fig. 5). Some subtypes are more closely related than others and therefore with minimal MinION data it is possible that misattributions can occur in the BLASTn screening step as seen in this study with 7.9% of reads generated from the H16 sample attributed to the close relative H13 (Table 2). Methods 1, 2 and 3 presented here successfully classified the HA subtypes within their respective clades (Fig. 5). Method 1 CAP3 de novo assembly was preferred over Method 2 and 3 due to the speed (30 min vs. 6 h vs. 4 h respectively). The success of this method highlights the need for further processing of the BLASTn analysis as it removes any reads that have been potentially misattributed.

The secondary analysis step involving Methods 1, 2 and 3 presented here (Fig. 1) successfully classified the HA subtypes within their respective clades (Fig. 5). Although Method 1 CAP3 generated the lowest mean percentage identity comparison between the MinION and Illumina sequences of 94% (Table 3) this percentage sequence identity was much higher than the 70% sequence divergence observed between AIV subtypes^54^. It is therefore unlikely that misattribution of HA subtypes will occur using this method. By adding the basic secondary analysis consensus step of Method 1 CAP3 to the pipeline presented here, any lack of confidence from the BLASTn analysis can be easily removed. When combined with phylogenetic alignment to local and international strains, the fast and accurate classification of the AIV subtypes is readily achievable. The ability to perform this classification in-field and in a timely fashion highlights the utility of the methods presented here. The aim of this study was not to reach perfect identity and coverage of the HA segment, but to confidently detect and subtype AIV in-field in the most rapid manner as this information informs incursion response decisions.

Despite the advances reported here for the in-field detection, sequencing and subtyping of AIV strains, there are further improvements that would facilitate faster sample processing of field samples. Advances in datasets and software applications could enable specific AIV sequence assemblers to be developed and trained on country or region-specific genome sequence data, for the specific identification of the HA subtype. The use of neural networks in genomics is also allowing a greater processing of data in a comprehensive and timely manner^55^. With suitable training, a neural network would overcome the inherent variability from the HA gene. A subsequent analysis would determine the origin of all AIV genome segments, including the HA gene, thus giving comprehensive data from the detected AIV in regional field settings, allowing determination of the origin of the virus and any biosecurity implications.

The in-field syringe-based extraction, although comparable to laboratory-based methods, was time consuming and unable to process multiple samples simultaneously. With the rising interest in viral genomic epidemiology, high throughput extraction methods are needed, and some advances are being made in this area. For example, QuickExtract^56–58^ has been successfully used in the extraction of SARS-CoV-2 for bulk RT-PCR screening but it has yet to be tested in a field capacity.

Enrichment of the target nucleic acid to be sequenced could be improved to enhance the ease and efficiency of sequencing targeted organisms and loci. In this study PCR was used to amplify the AIV genome prior to sequencing. However, the time required to amplify using PCR in-field took hours not minutes, which is a significant advance but still not optimal. Improved polymerase enzymes would remove these limitations and methods using the loop-mediated amplification^59^ (LAMP) *Bst* enzyme should be investigated. LAMP as an enrichment method could be deployed more widely as it has been proven to be effective in-field on viruses such as Dengue^60^, Chikungunya^61,62^ and Zika^63^. An alternative to enzymatic enrichment is via a more targeted sequencing approach. Adaptive sequencing approaches being developed by ONT enable the user to dictate which nucleic acid sequences are processed through the ONT sequencing platform^64,65^. By using raw reads, Payne et al.^65^ demonstrate the utility of sequencing selected reads on large human datasets and enriching for specific chromosome sequences. Similarly, Martin et al.^64^ have used adaptive sequencing effectively on mock bacterial communities which showed enrichment up to a fourfold increase. This method has the potential to enable enrichment of the desired sequence without the issues associated with enzymatic amplification, increasing the specificity of the sequencing platform.

Bioinformatics is an ongoing issue with in-field sequencing but hopefully as MinION in-field work increases and the importance of fast diagnostics becomes increasingly prevalent, more work will be dedicated to filling in the caveats of field research identified here and in other studies. Having a standard MinION based protocol for all viruses would be valuable, however the bioinformatics needs to be carefully crafted to the question at hand. Protocols such as the ARTIC networks’ RAMPART prove to be useful in constructing and identifying SARS-CoV-2 genomes in a real-time manner but when applied to viruses such as AIV they are not nearly as applicable as the number of reference genomes needed are much greater than those for the coronaviruses or Ebola to accurately encompass the diversity present. As sequencing becomes increasingly essential and routine in a broader number of scenarios, the data sets developed will enable a more data rich future leading to the need for ever faster diagnostics and methods, that the study has tried, in part, to address.

## Conclusions

This study developed an in-field detection and sequencing methodology for AIV and overcame all of the challenges faced to deliver outcomes that have the same level of similarity and accuracy as compared to the gold-standard Illumina IRMA constructed genome. The in-field detection and sequencing methodology applied in this study to historical AIV positive samples shows a high level of similarity and accuracy when compared to the gold-standard Illumina IRMA constructed genomes. However there remains significant challenges regarding in-field AIV detection and sequence analysis in both procedural and bioinformatic processes to deliver fast real-time, in-field sequence characterisation of AIV.

## Supplementary Information

Supplementary Information 1.

Supplementary Information 2.
