# Supplementary material for: Rapid, in-field deployable, avian influenza virus haemagglutinin characterisation tool using MinION technology
Source: Sci Rep. 2022 Jul 13;12:11886. doi: 10.1038/s41598-022-16048-y (PMC9279447; doi:10.1038/s41598-022-16048-y)
Supplement: Supplementary file 1 — Supplementary Information 1. [file 41598_2022_16048_MOESM1_ESM.docx]

**Supplementary Data 1**

Consumables taken into the field:

G-block diluted to 100 copy number
2x MinION R9.4 flow cell
Rapid barcoding and sequencing kit
Flow cell priming kit
Ice box + ice
AgPath Kit (including buffer and enzyme)
Influenza primer/probe TaqMan mix
MBTuni 12/13 primers 10µM
Superscript III buffer and enzyme
Biomeme M1 extraction kit (aliquoted out)

Dell computer
MIC PCR

Camping table
Eppendorf tubes
MIC tubes
Markers
Tube racks
Pipette rack
Pipettes (P1000, P200, P20, P10)
Pipette tips (P1000, P200, P20, P10)
Tip box
